# Mid-term outcomes of tantalum cup– a single centre study

**DOI:** 10.1186/s42836-021-00099-z

**Published:** 2021-10-09

**Authors:** Rajesh Bawale, Baseem Choudhry, Srinivasa Samsani

**Affiliations:** 1 Department of Trauma and Orthopaedics, Medway Maritime Hospital, Windmill Road, Gillingham, ME7 5NY UK; 2 Chigwell, UK

**Keywords:** Hip joint, Revision arthroplasty, Reconstruction, Complications

## Abstract

**Introduction:**

The cementless acetabular implants are commonly used in primary and revision hip arthroplasty. Reconstruction of acetabulum in case of bone defects can be challenging. The aims of this single center study are to review the mid-term outcomes of porous tantalum cups (TM) and evaluate complications.

**Methods:**

The midterm outcome of a trabecular metal tantalum modular uncemented cup was evaluated in 59 hips in 58 patients. In our group, we had 23 males and 35 females. The mean age was 70.11 years (range, 30 to 87 years). Four patients were lost to follow-up and 13 died during the period without having further surgeries attributed to the hip arthroplasty. The remaining 41 patients (42 revision hip arthroplasties) had complete data available.

**Results:**

The mean follow-up was 87 months, ranging from 24 to 144 months. Standard pelvic anteroposterior (AP) radiographs were used to assess and preoperatively classify acetabular defects *as per* Paprosky classification. The serial radiographs showed excellent stability, bone opposition and graft incorporation. Four patients had further surgeries. Two of these were due to infection (one superficial and one deep infection). One of the patients had washout and then removal of metal work, the other patient only had a washout and symptoms settled. One patient had vascular compromise and went for surgery to stem the bleeding. One patient had re-revision due to stem loosening and hence required surgery but the revision cup remained stable. We noted a 96% survival at an average of 7.2 years follow-up.

**Conclusion:**

The mid-term results with the trabecular metal cementless cup appeared to be promising in both primary and revision hip arthroplasty, even in the presence of considerable bone loss which requires bone grafting and augments.

**Level of evidence:**

IV.

## Introduction

In twenty-first century, total hip arthroplasty (THA) is a highly successful procedure that is being performed with increasing frequency. Increased incidence of primary THA is accompanied by a corresponding increase in revision THA along with associated concerns of diminished bone quality, bone loss and soft tissue compromise [1]. The population demographic is getting older and, increasing number of patients are re-presenting to arthroplasty surgeons for revision due to various pathologies. This has been projected to double by the year 2026 [2]. The complex hip pathology poses a challenge to the planning of primary hip arthroplasty. Cemented acetabular cups in primary total hips are shown to have variable survival rates than cementless cups [3, 4]. The utilisation of cup cage constructs, augments for large defects reduced the loosening rates up to 14% at 6 years follow-up. The introduction of porous metal implants with a range of accessory porous coated augments, shims and buttresses has led to further improvement in revision THA outcome.

The etiology of acetabular implant failure includes aseptic loosening, infection, instability, wear, trauma and osteolysis. Irrespective of the cause, revision surgery leaves pelvis with a significant bone loss which make further surgery more difficult.

The principles for addressing the periprosthetic bone loss are to augment bone stock, restore hip centre of rotation and offset, match limb lengths and deliver long-term implant stability to provide a functional, pain-free hip. Despite multiple options, acetabular revision remains a challenging problem but a small-sample single centre study and hip registries suggested uncemented tantalum cups have promising results in early to medium term [5, 6].

The porous metal implants have either titanium or tantalum porous coated metal surface and with press-fit implantation. It provides a stable mechanical surface between implant and host bone in the short term (primary stability), and supports osseointegration in the mild and long term [7, 8]. Tantalum (TM) has emerged as a viable alternative for acetabular reconstructions. It has a high elasticity between 2.5 and 3.9 MPa, working like subchondral bone to prevent stress shielding and allowing for physiological transfer of load to host bone, and a high friction coefficient which allows the implant to grasp bone-deficient acetabulum allowing for primary stability essential in revision arthroplasty [7, 8]. It also exhibits a very good prothrombogenic potential which promotes hematoma formation that is essential in early phases of bone healing and the risks of fracture and graft resorption are minimised [9].

The immunochemical study and in vitro biocompatibility test showed the TM has excellent growth, cellular adherence and abundant extracellular matrix formation on porous tantalum structures compared to porous titanium control [10]. These results suggest that porous tantalum implants can promote early and enhanced biologic fixation. Tantalum has an excellent biocompatibility, its ability to form a self-passivating surface oxide layer leads to the formation of a bone-like apatite coating resulting in excellent bony ingrowth allowing rapid and substantial bone attachment [11]. Tantalum components are associated with a lower incidence of failure and infection especially when used in infected hip arthroplasty revision cases [12]. Porous surfaces are intended for better osteointegration, and clinically and radiologically tantalum has shown better results against the titanium cups [13]. These properties of tantalum cups have gained priority over other revision cups available for revision at our institute.

The aims of this single centre study are to review the mid-term survival of porous tantalum cups and to evaluate complications.

## Methods

The data were collected from 2006 to 2018. A total of 59 acetabular revisions in 58 patients were performed using a TM acetabular component (Trabecular Metal™ Zimmer Biomet, Warsaw, IN, USA) by a single surgeon at our institution. Data were collected retrospectively and prospectively. The retrospective data were collected by using the patients' records, PACS systems for Images and outpatient clinic letters. The prospective data were collected when patients were investigated, diagnosed and treated. No differences in the data were noted.

A total of 245 acetabular revisions were done in our institution during this period by using cemented and uncemented cups available at our institution. The tantalum porous coated shell was used on the basis of the patients' background, the preoperative radiographic findings, the bone loss and the indications for revision surgery.

In our study group, we had 23 males and 35 females. The mean age was 70.11 years (range, 30 to 87 years). Four patients were lost to follow-up and 13 died during the period without having further surgeries attributed to hip arthroplasty. The remaining 41 patients (42 joints) who underwent revision hip arthroplasty had complete data available. All patients were postoperatively clinically and radiologically evaluated, 6 weeks, 6 months and then annually after the operation.

Both porous coated titanium and tantalum shells were available. The tantalum shell was chosen due to its superior properties over tantalum in revision settings [5, 13].

**Fig. 1:**
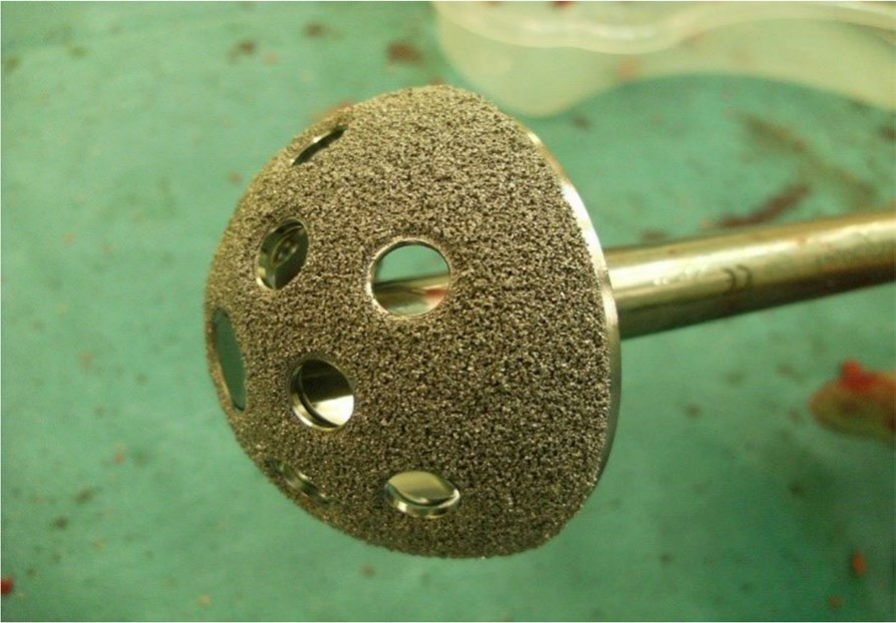
Tantalum shell

The patients underwent revision surgery by using uncemented trabecular TM acetabular component (Fig. 1). The size of cups used varied from 48 to 56. The screws and augments were used *as per* the indications. The preoperative planning was done in all the cases for (1) tantalum cup with bone graft and screws and (2) tantalum cup with augments. We used augments in 2 cases.

Thorough intraoperative bone loss assessment was carried out by the surgeon and the autograft/allograft was used to address the defects. The morselised bone allograft was used in 5 patients for autograft was not sufficient. The unconstrained acetabular liners were used in all cases. The head size was matched to fit the acetabular cup to provide the most appropriate stability whilst not compromising the range of motion.

All revisions were performed by the senior author using Southern Moore approach. Most revisions used an incision through the previous scars but if the anterolateral approach was used for the index procedure, a new incision was made to keep with the author’s approach. After achieving adequate exposure and removing the previous component, the periprosthetic membranes and bone tissue were taken and sent for histological and microbiological examinations. The defect and the remaining acetabulum were reviewed and decision was made to use a suitable porous tantalum shell. Then, a graft was used and impacted to fill the defects. After implantation and fixation of the augment, the augment was impacted and secured with additional screws. Postoperatively, full-weight bearing was advised and patients were reviewed in outpatient department at 6 weeks and at regular interval as indicated. The Oxford hip scores were used to measure pre- and postoperative results for all 42 hips (41 patients).

Patients attending clinic were reviewed for LLD discrepancy, complications such as wound infection, deep vein thromboembolism or pulmonary embolism, bleeding, nerve injury and, dislocation. Those who were not able to attend were contacted via phone or correspondence. Patient records were used to ascertain date of index surgery, date of revision surgery, age at revision, perioperative complications such as neurovascular injuries, number of dislocations and further re-revision surgeries. Standard pelvic anteroposterior (AP) radiographs were used to assess and preoperatively classify acetabular defects *as per* Paprosky classification. Latest radiographs were reviewed by two authors (BC, RB) for osseous integration, subsidence and lucencies (Table 2).

Primary end point was re-revision surgery for any reason. The data were collected for indications, the preoperative Paprosky grades, pre- and postoperative OHS and the postoperative complications. Kaplan-Meier analysis was used to assess tantalum cup implant survival. The revision surgery was used as an endpoint and deaths, loss of follow-up were censored. All *P*-values ≤0.05 were set as the level of statistical significance and two tailed *t*-test was used to assess the statistical difference. The data analysis was performed using MedCalc (MedCalc Software Ltd. Belgium) and Windows SPSS software.

## Results

In our study group, we had 23 males and 36 females. The mean age was 71 years (range 56–88 years). The aseptic loosening (60.3%) of the cup was one of the main indications for the revision surgery, followed by femoral aseptic loosening (11.8%), fracture (11.8%), cup-malalignment (8.8%), infection (3.3%) and metal-on-metal related pathology (4%) as shown in Table 1. Paprosky grading showed approximately 52% of the patients had grade I defects and 48% were rated grade II (Table 2). The mean follow-up time was 7.2 years (range, 2–12 years).

**Table 1:** Indications for revisions

Revision Indications	Numbers (*n*)	Percentage (%)
Aseptic Loosening	35	60.3
Femoral Loosening	7	11.8
Fracture	7	11.8
Cup Mal-alignment	5	8.8
Infection	2	3.3
Metal-on-metal related pathology	3	4

**Table 2:** Preoperative Paprosky Grades

Paprosky Grades	Numbers (*n*)	Percentage (%)
Grade I	31	52.5
Grade IIa	2	3.3
Grade IIb	10	17
Grade IIc	16	27.2

**Fig. 2:**
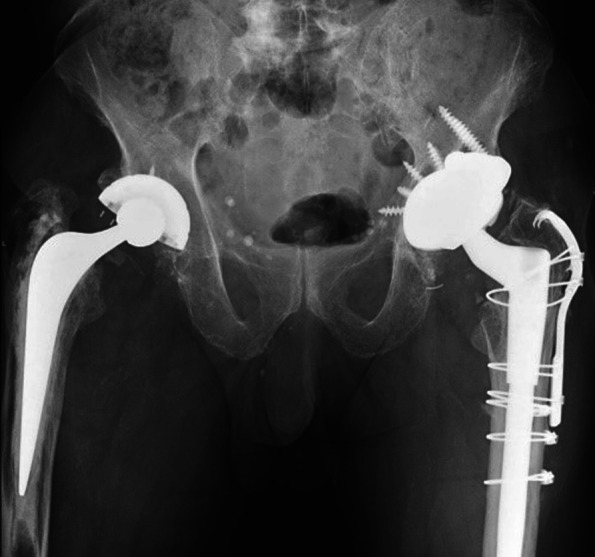
TM cup with augments and screws

**Fig. 3:**
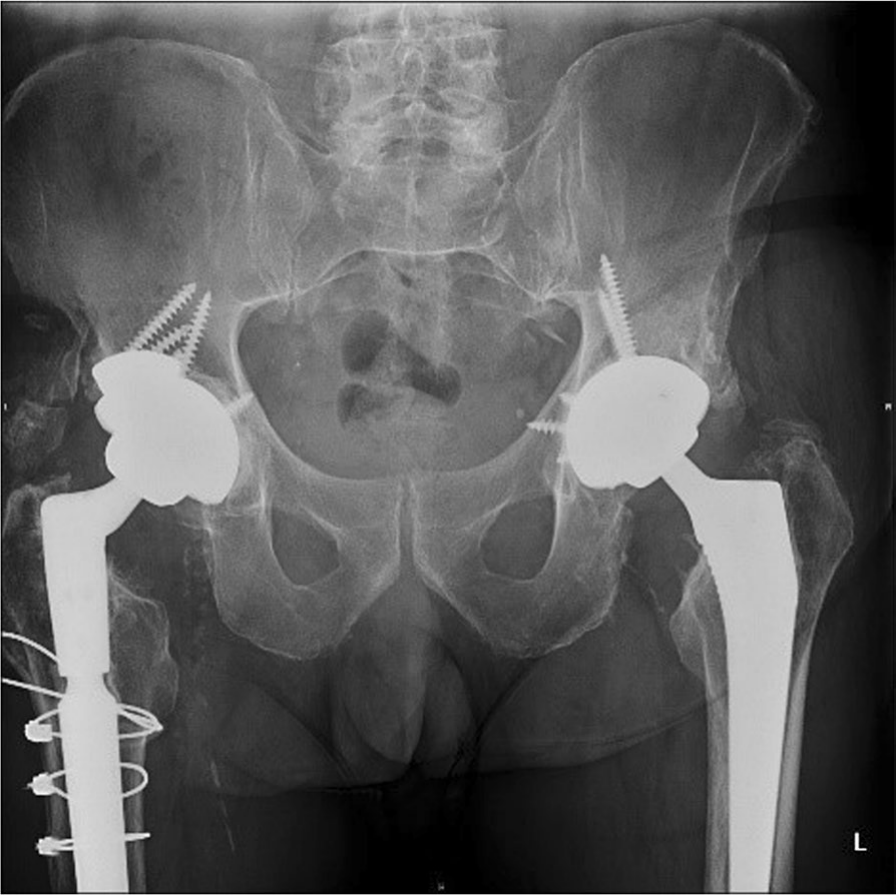
TM cup with good osteointegration

The host bone contact was noted to be equal or more than 60%. We noted bone loss in the superior acetabular segment in two cases which needed use of augment (Figs. 2 and 3). The screws were used in most of the cases to achieve the stability and all the images showed good integration of tantalum shells (Fig. 3).

**Fig. 4:**
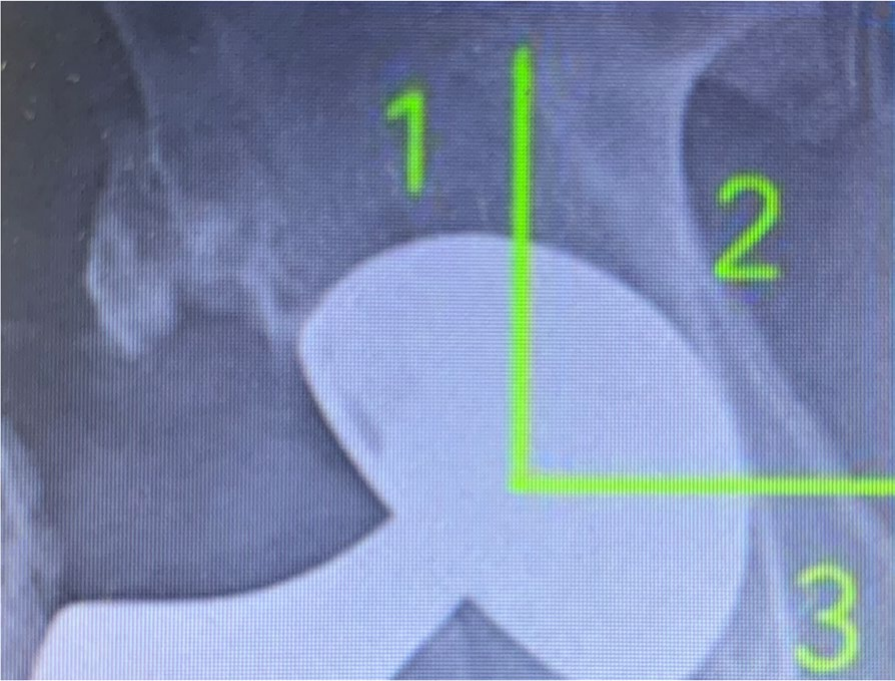
Delee and Charnley zones

**Fig. 5:**
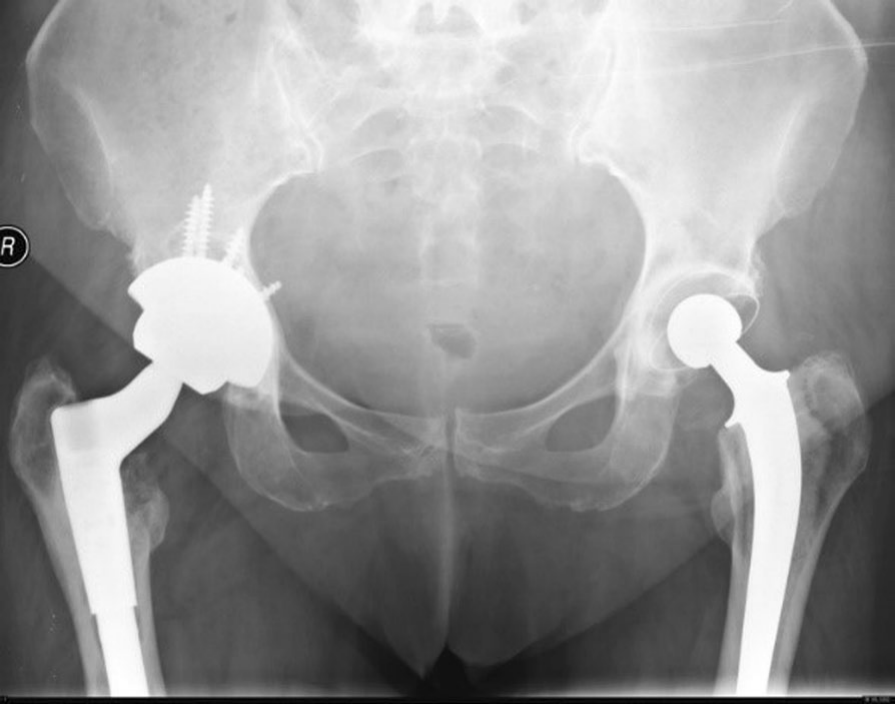
TM cup with Lucency in Delee and Charnley zone I

We did not notice any lucencies in the periacetabular areas. The radiographs show good bone integration in 38 out of 42 cases. However, we noted lucencies in the DeLee and Charnley Zone I in 4 cases and they were closely monitored and no further deterioration was noted (Figs. 4 and 5).

**Fig. 6:**
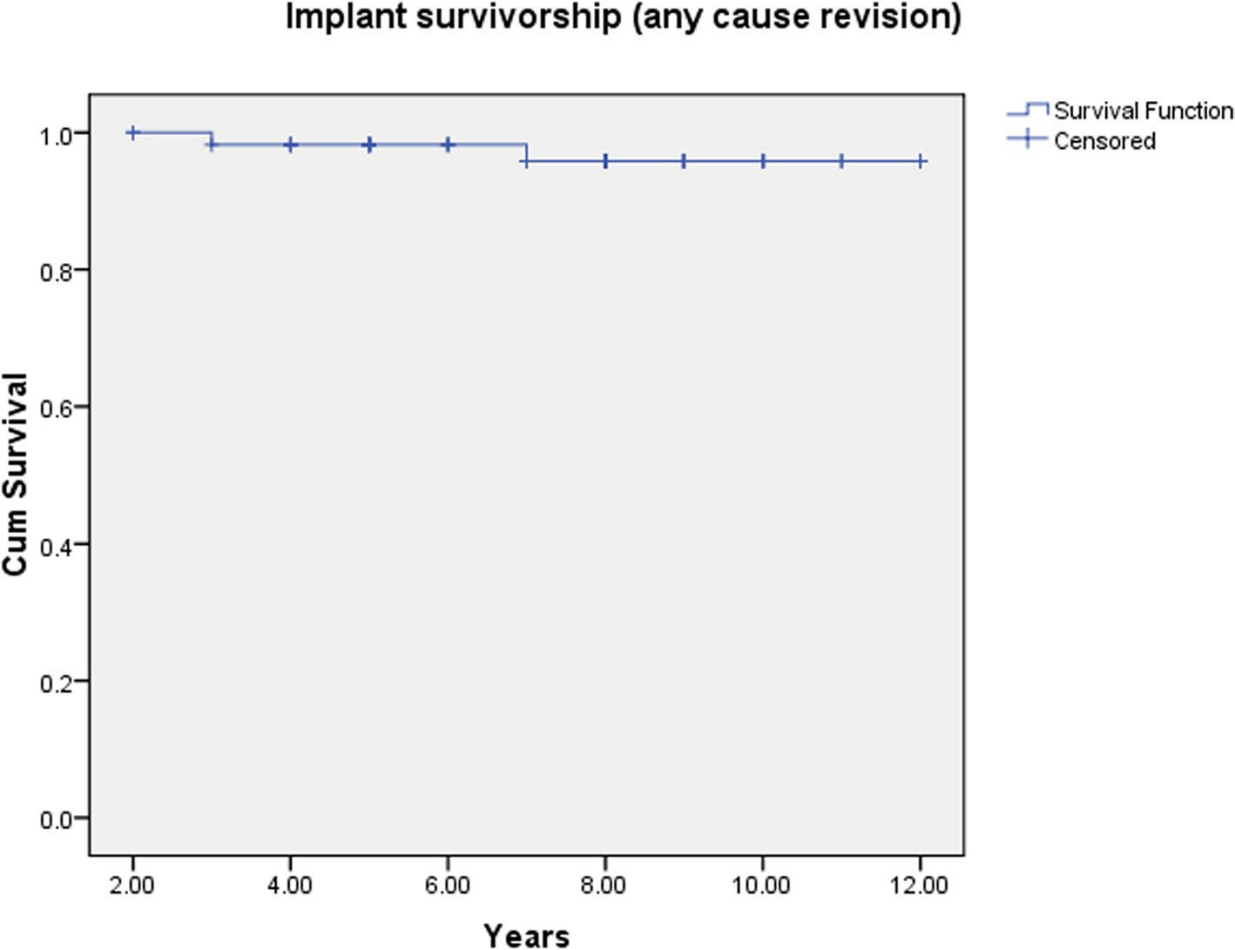
Survivorship – Kaplan Meir Curve

The Kaplan-Meier survival analysis curve showed a cumulative survivorship of 96% survival at 7.2 years (SE 0.138; 95% CI, 6.992–7.111) indicating one case of acetabular component revision due to infection (Fig. 6). At an average 7.2 years, this 2 to 12-year group had 96% survivorship of the acetabular component.

The clinical and radiographic assessments of porous coated tantalum acetabular components support good outcomes and suggest very successful mid-term results. The mean preoperative Oxford Hip score was 27.2, which improved to  a mean score of 33.32 postoperatively at 24 months and two tailed *t*-test showed *P* value < 0.00001. We did not notice any drop outs during follow-up.

### Complications

Table 3 illustrates the postoperative complications. In our cohort, 3 patients had further surgeries. Two of these patients received the operations due to infection (one deep and one superficial infection). The patient with deep infection had a delayed presentation 4 years after surgery and underwent staged revision of the cup. The superficial infection was diagnosed 4 weeks postoperatively and was managed with the debridement and washout followed by the antibiotics. One patient had re-revision due to femoral stem aseptic loosening and hence required surgery but the cup remained stable and was not revised. One patient had vascular compromise and was treated by a vascular team to stem the bleed. We did not see any dislocation in our study. Three patients (5%) had the limb length issues (less than 2 cm), which were addressed by orthotics. One patient was diagnosed with deep venous thrombosis below knee, which was treated *as per* the local treatment protocol. We did not observe any dislocations in this study.

**Table 3:** Post-revision complications

Complications	Numbers (n)	Percentage (%)
Infections	2	3.2
NV Injury	1	1.6
DVT/PE	1	1.6
LLD Felt	3	5
Stem loosening	1	1.6
Dislocations	0	0

## Discussion

Aseptic loosening (42.9%) of the THA components has been reported as the major reason for THA revision [1, 14, 15] and the loss of bone with associated bone defects poses a significant surgical challenge [16, 17]. Revision THA has a greater chance of failure than the primary THA due to compromised soft tissues, bone loss and complexity of the procedure. The plasma-sprayed titanium acetabular cups allow bone ongrowth rather than bone ingrowth [18] and have been associated with poor integration with the sclerotic host bone, increased bone resorption and increased difficulty with future revision procedures [19]. Porous coated uncemented acetabular components provide press-fit implantation to achieve adequate primary stability during surgery and early postoperative phase, and secondary stability through adequate osseointegration achieved later with bony ingrowth [20]. The tantalum cups and augments have been noted to have satisfactory clinical and radiological outcomes in patients who underwent THA due to severe bone defects of Paprosky type III or IV [21].

Porous trabecular tantalum has gained popularity over the last decade. This is because TM minimizes the stress shielding, has good ingrowth properties, which reduces the use of bone graft in revision, and promotes the osseous integration. Our study showed excellent outcomes of trabecular TM acetabular component in terms of the stability and fixation in the acetabulum revision hip arthroplasty with a 96% survival at 7.2 years. This was comparable to the result achieved by Mittien et al. [22]. Current literature has shown encouraging and comparable early-to-midterm results. We also noted few published studies with 5 to 10 year follow-up but our study is different from all these studies since we did not notice any dislocation and we  attained a 96% TM component survival at a mean follow-up time of 8.2 years [6, 23, 24]. We think that our surgical technique is the key factor responsible for reduceddislocation rate. The mean preoperative Oxford Hip score was 27.2, which improved to a mean score of 33.2 24 months after operation. The difference was statistically significant  for two tailed *t*-test showed *P* value < 0.00001.

The dislocation has been reported in the literature as one of the postoperative complications but we did not notice any dislocation in this study [6]. The use of trabecular metal™ shell has shown good mid-term outcomes in our study which was comparable with the similar studies [16, 25]. The use of augments with screws was noted to be  a very reliable way of addressing any acetabular bone defects, though we used them only in 1 case [13, 26]. Our study mainly aimed at addressing the mid-term outcomes for tantalum cups used mainly for acetabular revisions. However, the results are encouraging with primary hip arthroplasty [27]. We noted that one case of deep infection 4 years after the index procedure and needed 2-stage revision, which is consistent with the published literature [28]. The tantalum cups seem to provide good stability due to integration of the trabecular metal into the cancellous bone in irradiated bone [23]. The use of tantalum acetabular components during revision THA is associated with a lower incidence of infection and we noted similar findings in our study with an infection rate of 1.6% for deep infection [29]. We also noted that short- and mid-term outcomes with the use of tantalum porous coated shell are encouraging, but some of the studies recommended comparison be made in terms of the long-term outcomes between tantalum and titanium revision shell to further prove the tantalum superiority [30]. However,  most studies undoubtfully showed highly desirable outcomes with the use of porous tantalum (TM) cups, both in the mid-term and the long-term [31].

To sum up, in our study, we noted a 96% tantalum cup survival at an average of 7.2 years of follow-up, no dislocations, a 1.6% deep infection rate and a 1.6% superficial infection rate, which is more promising in comparison with other studies. Our study showed good survival with minimal complications.

One of the limitations of our study is that it was a retrospective study, which might cause biases. Moreover, our cohort of patients did not have a significantly high Paprosky grade and hence we cannot comment on the stability and outcomes in patients with large acetabular defects in our study.

## Conclusion

To conclude, the mid-term results in our revision arthroplasty cases showed that highly porous coated tantalum cups could achieve a good stability, caused less revision due to excellent integration of the trabecular metal to the cancellous bone, and had lower infection rate.

## Data Availability

Not applicable.
